# Colonic Mucosal Immune Activation in Mice with Ovalbumin-Induced Allergic Airway Disease: Association between Allergic Airway Disease and Irritable Bowel Syndrome

**DOI:** 10.3390/ijms23010181

**Published:** 2021-12-24

**Authors:** Sanghyun Kim, Bora Keum, Junhyoung Byun, Byoungjae Kim, Kijeong Lee, Jiwoo Yeon, Jaemin Lee, Hyuksoon Choi, Eunsun Kim, Yoontae Jeen, Hongsik Lee, Hoonjai Chun, Taehoon Kim

**Affiliations:** 1 Department of Internal Medicine, College of Medicine, Korea University, Seoul 02841, Korea; snell177@naver.com (S.K.); borakeum@hanmail.net (B.K.); jmlee1202@gmail.com (J.L.); mdkorea@gmail.com (H.C.); silverkes@naver.com (E.K.); ytjeen@korea.ac.kr (Y.J.); hslee60@korea.ac.kr (H.L.); 2 Department of Otorhinolaryngology-Head & Neck Surgery, College of Medicine, Korea University, Seoul 02841, Korea; wnsgud816@naver.com (J.B.); autru222@gmail.com (B.K.); snell177@hanmail.net (K.L.); snell1777@gmail.com (J.Y.)

**Keywords:** allergic airway diseases, irritable bowel syndrome, mast cells, salbutamol

## Abstract

Recent studies on the pathophysiology of irritable bowel syndrome (IBS) have focused on the role of mast cells (MCs) in intestinal mucosal immunity. A link between allergic airway diseases (AADs) and IBS has been suggested because both diseases have similar pathophysiology. We aimed to investigate whether the induction of AAD in mice could lead to inflammation of the colonic mucosa, similar to IBS. We also evaluated whether this inflammatory response could be suppressed by administering a therapeutic agent. Mice were divided into three groups: control, AAD-induced, and salbutamol-treated. An AAD mouse model was established by intraperitoneal injection and nasal challenge with ovalbumin. Mice with AAD were intranasally administered salbutamol. Analyses of cytokine levels, MC count, and tryptase levels in the intestinal mucosa were performed to compare the changes in inflammatory responses among the three groups. Inflammation was observed in the intestinal mucosa of mice in the AAD group. This inflammation in AAD mice was suppressed after salbutamol treatment. Our study demonstrates that AAD induces an inflammatory response similar to that in IBS, suggesting a possible association between IBS and AADs. In patients with IBS with such allergic components, salbutamol may have the potential to alleviate the inflammatory response.

## 1. Introduction

Irritable bowel syndrome (IBS) is a gastrointestinal disorder that manifests as recurrent abdominal pain associated with bowel movements or changes in bowel habits [1]. IBS is commonly diagnosed worldwide and has an estimated prevalence of 10–15%; however, its pathophysiology has not yet been accurately identified [2]. Although IBS was previously considered a psychosomatic or functional disease, it is now considered a heterogeneous group of conditions with various subsets. Among various diseases may cause IBS, it has been suggested that allergic diseases may be linked to IBS.

Recent studies on the pathophysiology of IBS have focused on the role of mast cells (MCs) in mediating intestinal mucosal immunity and low-grade inflammation [3,4]. Allergic airway diseases (AADs), such as allergic rhinitis (AR) or asthma, are type I hypersensitivity reactions caused by the release of mediators from MCs. It has been suggested that AADs may be linked to IBS, as both diseases share a similar pathophysiology and result from the activation of MCs [5]. Retrospective cohort studies have reported an epidemiological association between AADs, such as allergic rhinitis or asthma and IBS [6,7].

We assume that among the various subsets of IBS, some are associated with allergic components. In particular, we believe that AAD might be associated with the incidence and exacerbation of IBS in certain groups of patients. We hypothesized that the inflammatory reactions in the intestinal mucosa might be similar to those occurring after the induction of an airway inflammatory response. We aimed to ascertain if the activation of intestinal MCs in an AAD murine model triggers an inflammatory response similar to that in IBS.

As a secondary endpoint, we evaluated the potential of a therapeutic agent against AAD to suppress intestinal mucosal inflammation in mice with AAD. Salbutamol, a widely known first-line therapy for asthma, is a bronchodilator that relaxes the bronchial smooth muscles by selectively acting on the β2 receptor of the sympathetic nerve. It is also known to act as an MC stabilizer that reduces MC activation by blocking the secretion of MC mediators [8]. Salbutamol was administered nasally to mice with AAD, and changes in the intestinal mucosa were assessed. 

## 2. Results

### 2.1. Allergic Airway Mouse Model Establishment

We induced AAD in 20 male BALB/c mice and performed multiple tests to confirm that the allergic mouse model was successfully established. The nasal rubbing and sneezing symptom scores in the AAD group were significantly higher than those in the control group. Ovalbumin (OVA)-specific IgE levels were also significantly higher in the AAD group (27.1 ± 1.7 ng/mL) than in the controls (16.5 ± 0.3 ng/mL; *p* < 0.05) We sacrificed ten mice for evaluation of AAD group and treated the remaining ten mice with salbutamol as described in the Materials and Methods section.

### 2.2. Evaluation of Cytokine Expression in Colonic Mucosa of AAD Mouse Using Quantitative RT-PCR Method (qRT-PCR) 

**Figure 1:**
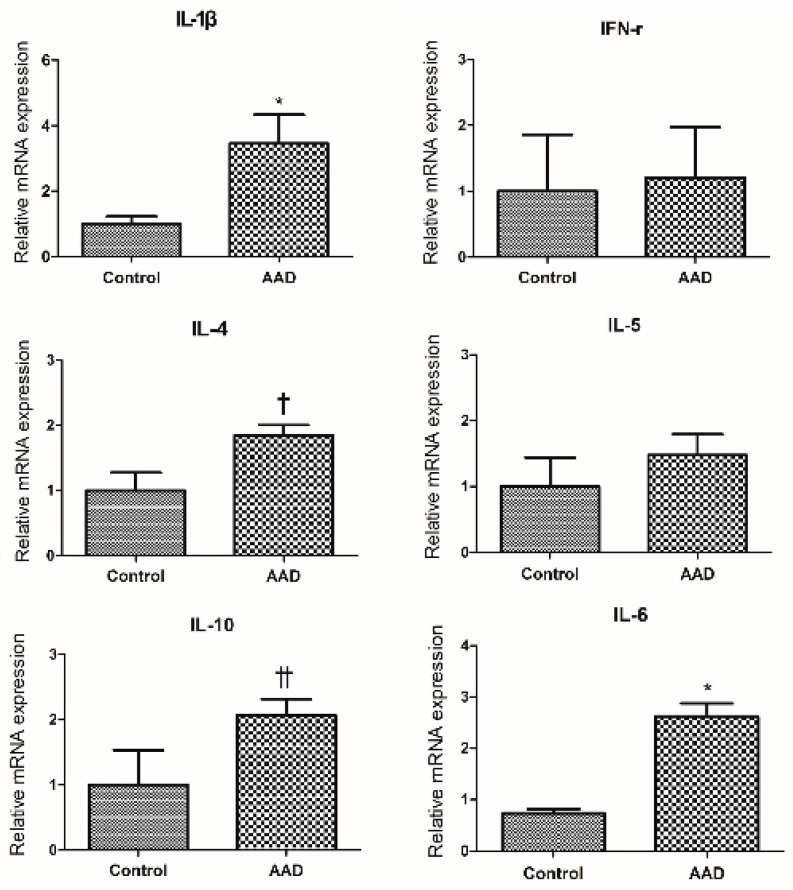
Comparison of cytokine expression between the allergic airway disease (AAD) and control groups by qRT-PCR. Interleukin (IL)-1β and IL-6 levels were significantly higher in the intestinal mucosa of AAD mice than in the control group (* *p* < 0.05). IL-4 and IL-10 levels showed an increasing trend in AAD mice († *p* = 0.058 and †† *p* = 0.067, respectively).

We investigated the expression of cytokines and their corresponding mRNAs in colonic mucosa of AAD mice. The qRT-PCR results showed that IL-1β and IL-6 levels were significantly higher in the intestinal mucosa of AAD mice than in the control group (*p* < 0.05; Figure 1). As shown in Figure 1, IL-4 and IL-10 levels showed an increasing trend in AAD mice (*p* = 0.058 and *p* = 0.067, respectively). No significant differences were observed in the expression of interferon (IFN)-γ and interleukin (IL)-5 between the two groups.

### 2.3. Macroscopic Scoring of Colon Changes

The macroscopic changes of the colon were investigated by measuring colon length, body weight, and stool characteristics in AAD mice and the group administered with salbutamol to AAD mice. A stool score index of the severity of colonic changes described by Kimball et al. [9] was used. AAD group showed colon shortening compared with control group (*p* < 0.05; Table 1). The feces were dark brown, solid masses in control mice; whereas AAD mice had bright brown colored watery stool, indicating diarrhea. The colon length of the salbutamol group did not show significant change compared to the control group. There were also no differences in colon weight and the stool score between the two groups. (Table 1).

### 2.4. Histopathological Analysis of the Colonic Mucosa

**Figure 2:**
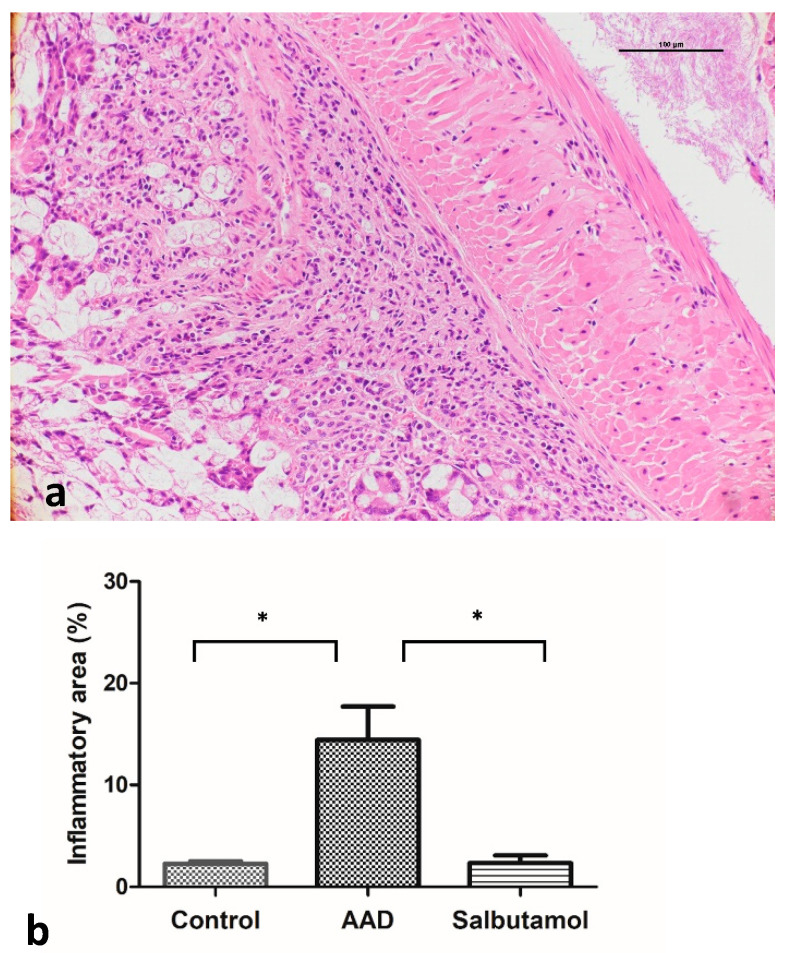
Microscopic analysis of the colonic mucosa. (**a**) Histological analysis of intestinal mucosa of ovalbumin (OVA)-challenged mice after hematoxylin and eosin staining (200×). Representative images depict inflammatory lesions in the intestinal mucosa. Erosions of the intestinal epithelia and infiltration of lymphocytes and neutrophils in the lamina propria and submucosa were observed. (**b**) The calculated area of the intestinal mucosa where inflammatory cells infiltrated was compared to the total mucosal area of the sample. The percentage of inflammatory area was compared among the groups. * *p* < 0.05.

Inflammation and epithelial cell degeneration were analyzed in hematoxylin and eosin (H&E)-stained colon sections of control and AAD mice. No morphological differences, such as inflammatory infiltration by lymphocytes, epithelial erosion, and crypt loss, were observed in the control and AAD groups (Figure 2a). The proportions of the inflammatory area of the groups were also compared with each other. A significant increase in the percentage of inflammatory area was observed in AAD mice (control group: 2.68% ± 0.26%; AAD group: 14.43% ± 3.29%; *p* < 0.05) (Figure 2b). Compared with the AAD group, the salbutamol-treated group showed a significant decrease in the extent of inflammatory lesions (AAD group: 14.43% ± 3.29%; salbutamol group: 2.33% ± 0.74%; *p* < 0.05).

### 2.5. Evaluation of Intestinal Cytokine Expression Using Enzyme-Linked Immunosorbent Assay (ELISA)

**Figure 3:**
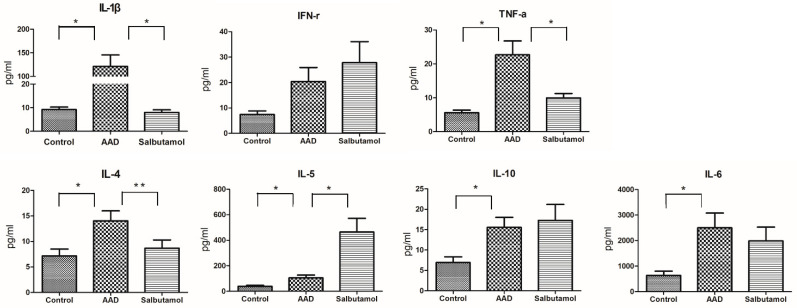
The results of cytokine ELISA assay. T-helper 1 (Th1) (IL-1β, interferon [IFN]-γ, and tumor necrosis factor [TNF]-α), Th2 (IL-4, IL-5 and IL-10), and proinflammatory cytokine (IL-6) levels were measured using ELISA with a multiplex protein assay kit. * *p* < 0.05, ** *p* = 0.064.

The localized expression of T-helper 1 (Th1) (IFN-γ, IL-1β, and TNF-α), Th2 (IL-4, IL-5 and IL-10), and proinflammatory cytokines (IL-6) in the colonic tissue was determined using the corresponding ELISA kits. The levels of IL-1β, TNF-α, IL-4, IL-5, IL-6, and IL-10 were significantly higher in AAD mice than in control mice (*p* < 0.05) (Figure 3). There were no significant differences in IFN-γ levels between the two groups of mice.

The levels of IL-1β and TNF-α were significantly lower (*p* < 0.05) in the salbutamol group than in the AAD group. IL-4 levels tended to decrease (*p* = 0.064). IFN-γ, IL-6, and IL-10 levels did not show any significant changes. 

### 2.6. Mucosal MC Counts

**Figure 4:**
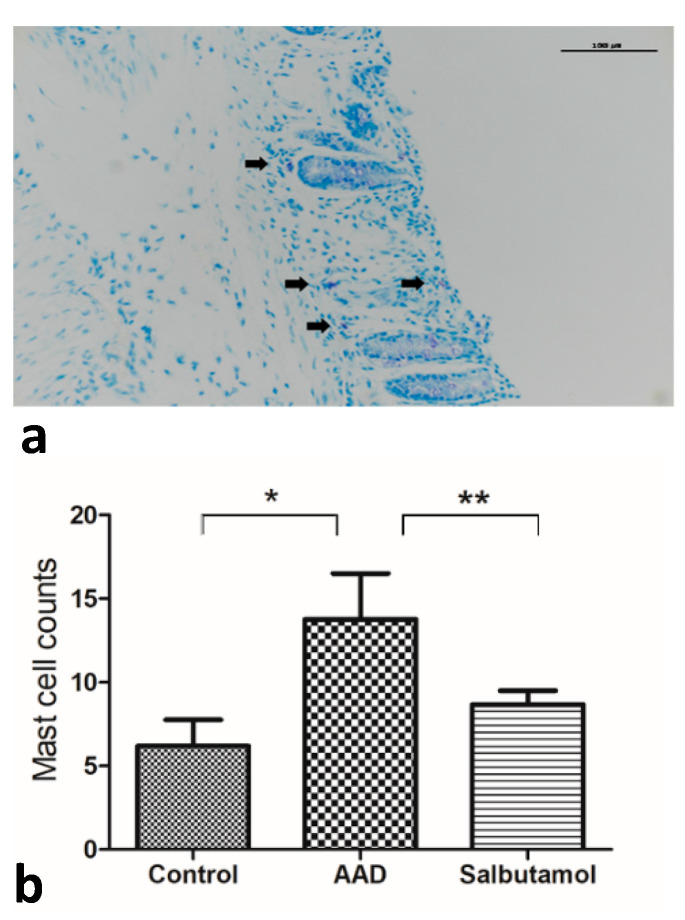
Evaluation of mucosal mast cells. (**a**) Toluidine blue staining of mast cells. (**b**) The number of mast cells in each group was counted and compared among the groups. * *p* < 0.05, ** *p* = 0.068.

Most MCs were observed in the submucosa rather than in the epithelium or lamina propria (Figure 4a). The number of MCs was markedly increased in the intestinal mucosa of AAD mice compared with that in the control group (control group: 6.20 ± 1.56; AAD group: 13.75 ± 2.75 (mean MC counts per high-power field [MC/HPF]); *p* < 0.05) (Figure 4b). The number of MCs tended to decrease in salbutamol-treated mice compared with AAD mice (salbutamol group: 8.67 ± 0.84 (MC/HPF); *p* = 0.068).

### 2.7. Tryptase Western Blotting

**Figure 5:**
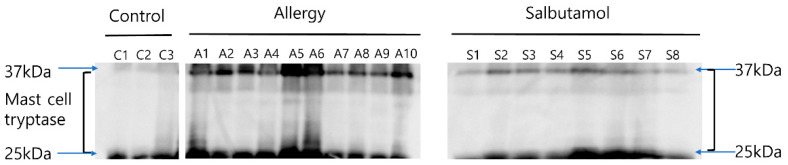
Representative western blot analysis of tryptase levels in murine intestinal mucosa. Bands were cropped from different parts of the same gel or from different gels. Three mice in the control group, ten mice in the AAD group, and eight mice in the salbutamol group were compared and analyzed.

Analysis of biopsy lysates by Western blotting revealed higher levels of tryptase in AAD mice (n = 10) than in control mice (n = 3) (Figure 5). The salbutamol group (n = 8) showed an overall decrease in tryptase expression compared with the AAD group.

## 3. Discussion

In this study, we evaluated whether inducing AAD in mice via an OVA challenge triggered an inflammatory response similar to that in IBS in the intestinal mucosa. We demonstrated that inflammatory responses occurred in the intestinal mucosa of AAD mice. We observed a significant increase in the relative length of inflammatory lesions in the colonic mucosa of AAD mice. The results of qRT-PCR analysis and ELISA of colonic tissues showed changes in the expression of various cytokines. Evidence of MC-mediated immune responses was also suggested. The number of MCs in the intestinal mucosa of the AAD group was significantly greater than that in the control group. Western blot analysis results revealed increased levels of tryptase in the intestinal mucosa of AAD mice. 

To date, accumulating evidence has shown the important role of MCs in the pathophysiology of IBS. Multiple studies have demonstrated that there is an increase in the number of MCs and their products, such as tryptase and histamine, in the intestines of patients with IBS [10,11]. Moreover, the activation and degranulation of MCs have been shown to influence the expression of IBS [12]. Additionally, a recent study has shown that close proximity between the intestinal nerve and MCs increases the severity of IBS symptoms [13].

MCs, which play crucial roles in various immune responses, are best described in the context of allergic diseases. The association between allergic diseases and IBS has only been discussed in the case of food allergies. However, recent studies have reported an increase in the prevalence of IBS in individuals with non-food allergies, suggesting that these two diseases may be linked [5]. Studies have established an epidemiological relationship between AADs, such as AR and asthma, and functional gastrointestinal disorders (e.g., IBS and functional dyspepsia) [7,14,15].

**Figure 6:**
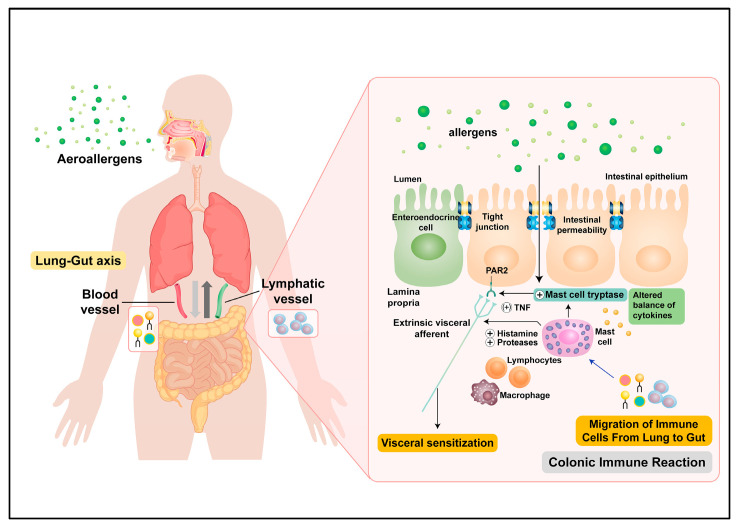
Overview of the pathophysiology of intestinal inflammation induced by aeroallergen. Proposed regulation of disease exacerbation by the lung-gut axis. When the lung immune system is disturbed, for example, during infection or allergen exposure, the intestinal immune system is altered as well by migration of immune mediators from lung to gut. Direct inflammatory reactions can also be induced in the intestine by the ingestion of aeroallergens.

The mechanism through which allergens passing through airways during AAD induce an inflammatory response in the intestine has not yet been elucidated. One possible mechanism is the remote activation of immune cells at distant mucosal sites. The mucosal immune system protects the host from foreign pathogens, such as allergens, toxic elements, and infectious microbes. Several studies have suggested that distinct mucosal sites may interact to protect the host from external pathogens. Research is underway on the interplay between different mucous membranes in the body. Particularly, it has been suggested that the lungs and gastrointestinal tract, which are the central organs responsible for primary defense, interact closely with external insults [16]. Studies have reported evidence of this lung–gut axis. Gastrointestinal allergies due to ingested foods are often preceded or accompanied by respiratory symptoms, such as sneezing, nasal congestion, coughing and wheezing [17]. Patients with asthma and allergic rhinitis have been shown to have an increased number of eosinophils in their intestinal tissues [18]. Brandt et al. demonstrated that OVA-induced gastrointestinal allergy not only enhances the upper airway responses to OVA, but also to other aeroallergens, such as house dust mites (HDMs) [19]. Ruane et al. showed that murine lung dendritic cells induce the migration of protective T cells to the gastrointestinal tract [20]. Collectively, these results suggest that allergic reactions at one mucosal site can affect distant mucosal sites. Immune-inflammatory responses in the intestine may worsen or induce subsequent immune-inflammatory responses in the upper respiratory tract, or vice versa (Figure 6).

Direct inflammatory reactions can be induced in the intestine by the ingestion of aeroallergens, such as HDMs, pollen, mold and animal dander [21]. Most aeroallergens are expelled via coughing, but a small amount passes through the gastrointestinal tract. Moreover, there have been cases where allergens have been shown to contaminate food. Mice that were intranasally injected with extracts of HDMs, *Aspergillus fumigatus*, and cockroaches displayed eosinophilic esophagitis [22]. Exposure to birch pollen has been shown to induce local duodenal inflammation and lead to increased numbers of eosinophils and IgE-carrying MCs [23]. Studies have shown that patients with IBS are more sensitive to aeroallergens and that patients with atopic IBS have higher levels of total serum IgE and more severe aeroallergen allergy than patients with non-atopic IBS [24]. These results suggest that ingested aeroallergens can be an environmental trigger in IBS pathogenesis (Figure 6).

Owing to the emphasis on the role of MCs in the pathophysiology of IBS, various MC-targeting drugs have been tested. Drugs with numerous mechanisms of action that inhibit the activation, maturation, and development of MCs as well as the homing of MCs to the gut have been tested. Disodium cromoglycate (DSCG) and ketotifen are classical MC stabilizers and are the most widely studied drugs. In a previous study, DSCG suppressed visceral hypersensitivity during colon dilatation in stress-sensitive mice [25]. In another study, ketotifen prevented intestinal hypermotility and mucosal MC hyperplasia in animals [26]. However, in human studies, neither drug yielded satisfactory results. DSCG reduced the release of tryptase from jejunal biopsies in small-scale preliminary studies [27]. Ketotifen improved IBS symptoms and quality of life but did not prevent MC hyperplasia or activation in patients with IBS [28]. Several research groups have shown that β2-adrenoceptor agonists inhibit the release of histamine, prostaglandins, and pro-inflammatory cytokines from human MCs following IgE stimulation. Russo et al. demonstrated that the intranasal administration of salbutamol inhibits histamine and tryptase release, supporting the hypothesis that salbutamol may exert a protective effect by inhibiting MC activation [8]. However, the function of β2-adrenoceptor agonists in intestinal MCs has not been comprehensively studied. In the present study, AAD mice were treated with salbutamol to evaluate whether the drug suppressed intestinal MC activity. When AAD mice were treated with salbutamol, inflammatory markers, such as tryptase and cytokine levels and MC counts, decreased significantly. Taken together, these data indicate that salbutamol may suppress the inflammatory response in AAD mice.

Our study has several limitations. First, it may be questioned whether the inflammatory response induced in the intestine is secondary to the immune-inflammatory response induced in the upper respiratory tract. Furthermore, the possibility that the OVA solution, which was used for nasal OVA stimulation, flows into the alimentary tract cannot be ruled out. Nasal OVA instillation was performed in small portions to ensure that the OVA drops did not flow into the mouth.

Second, the similarity between the inflammatory changes in the intestine of AAD mice and IBS is not clear. Although we observed changes in inflammatory cytokines in the present study, there was no robust evidence indicating that these changes were similar to those observed in IBS. Various studies have reported altered cytokine expression levels in patients with IBS. Changes in the expression of inflammatory cytokines in the intestinal mucosa of patients with IBS are ambiguous [1]. The expression of pro-inflammatory cytokines (IL-1β, IL-6, IL-8 and IL-12) and activated lymphocytes (IL-2 and IFN-ɣ) have been found to be either upregulated [2,3] or downregulated [4]. Increased MC counts and tryptase levels have been consistently reported in the colonic mucosa of patients with IBS in previous studies. Similar results were observed in the present study.

## 4. Materials and Methods

### 4.1. AAD Mouse Model

**Figure 7:**
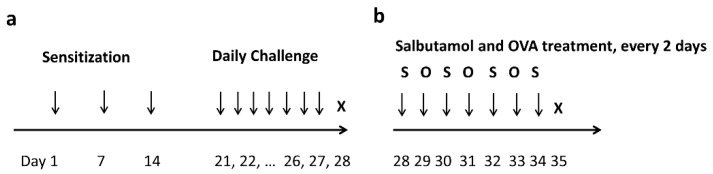
Protocol for the induction of AAD and salbutamol treatment in mice. (**a**) The timeline illustrates that mice were intraperitoneally injected with OVA, alum, or saline and subsequently intranasally administered saline or OVA from days 21 to 27. Mice were sacrificed on day 28. (**b**) After completion of the nasal OVA challenge, mice from the salbutamol group were treated with salbutamol. Mice were intranasally challenged with salbutamol and OVA every alternate day from days 28 to 34. Mice were sacrificed on day 35.

All procedures were approved by the Institutional Animal Care and Use Committee of the Korea University College of Medicine (approval number: KOREA-2019-0141). All experiments were conducted in accordance with the National Institutes of Health Guide for the Care and Use of Laboratory Animals, ARRIVE guidelines, and other relevant guidelines and regulations. Eight-week-old female BALB/c mice (Orient, Daejeon, Korea) were maintained in pathogen-free conventional animal facilities. A total of thirty mice were randomly divided into two groups (20 and 10 mice in the AAD and control groups, respectively). An OVA-induced AAD murine model was established using a previously described method [29]. Mice were sensitized with an intraperitoneal injection of 25 μg OVA (Sigma-Aldrich, St Louis, MO, USA) suspended in 1% aluminum hydroxide (Thermo Fisher Scientific, Waltham, MA, USA) on days 1, 7 and 14. On days 21–27, the OVA-sensitized mice were intranasally challenged with 50 μL of OVA (10 mg/mL) mixed with saline (Figure 7a). The control group was sensitized and challenged with saline instead of OVA. Intranasal challenge with OVA was performed by holding the mouse in a supine position and instilling a drop under the nose. Measures were taken to ensure that OVA was not aspirated into the gastrointestinal tract.

### 4.2. Macroscopic Evaluation of Inflammation

Ten mice from each of the control and AAD groups were humanely sacrificed by cervical dislocation 24 h after final stimulation. After sacrifice, the colon (distal to the cecum) was removed, and its weight and length were measured after removing fecal contents. Stool condition was scored by three researchers in a blind manner; stool score (0, normal; 1, loose/moist; 2, amorphous/sticky; and 3, diarrhea). The colons were divided into halves by longitudinal dissections, and one-half of the colonic tissue was immediately preserved in liquid nitrogen for protein assay and subsequent RNA extraction. The other half was rolled up from the distal to the proximal end to form a “Swiss roll.” Specimens were fixed in 4% PFA (Carl Roth, Karlsruhe, Germany).

### 4.3. qRT-PCR for Inflammatory Cytokines in the Intestinal Mucosa

qRT-PCR was performed on colonic mucosal tissues to determine whether there was a change in the expression of inflammatory cytokines after AAD induction. The localized expression of Th1 (IFN-γ IL-1β), Th2 (IL-4, IL-5 and IL-10), and proinflammatory cytokines (IL-6) was evaluated. Total RNA was isolated from 100 mg of colonic tissues using an RNA Extraction Kit (Takara, Shiga, Japan) and processed with the PrimeScript RT Reagent Kit (Takara, Shiga, Japan) to synthesize cDNA. mRNA was purified by treatment with chloroform, isopropanol and ethanol. cDNA was synthesized from RNA using the cDNA Synthesis Master Mix (GenDEPOT, Katy, TX, USA). cDNA was amplified and quantified using SYBR Green Master Mix (Qiagen, Hilden, Germany). The primer sequences are listed in Supplementary Table S1 qRT-PCR was performed in triplicate for each sample using a real-time thermal cycler system (TP850; Takara, Shiga, Japan). The reaction conditions for the amplification of DNA were as follows: 95 °C for 120 s and 50 cycles at 95 °C for 15 s and 60 °C for 45 s. Target mRNA expression was normalized to that of glyceraldehyde 3-phosphate dehydrogenase. Data were analyzed using the ΔCt method.

### 4.4. Salbutamol Treatment after OVA Challenge

The salbutamol group was prepared by administering salbutamol to remaining ten AAD mice. The experiment was performed by dispensing salbutamol (50 µM) and OVA into the nasal cavity every alternate day, starting from day 1 after the successful establishment of the allergy model (Figure 7b). Ten salbutamol-treated mice were sacrificed on day 35. Inflammatory changes in AAD group, salbutamol group, and control group were analyzed. 

### 4.5. Histological Evaluation of Inflammation

H&E staining was performed on paraffin-embedded 4 mm thick sections of the Swiss roll specimens of the colon. For the characterization of histopathological changes, such as inflammation, crypt distortion, and loss of goblet cells, all H&E-stained sections were examined by two independent pathologists in a blinded manner using an Olympus BX51 microscope (Tokyo, Japan). Based on a previously described method, an inflammatory score ranging from 0 to 3 (0 = none, 1 = mild, 2 = moderate, and 3 = severe) was assigned [30]. Inflammatory area (%) was defined as a proportion of area invaded by inflammatory cells compared to the total mucosal area. The area of inflammatory lesions was calculated in four contiguous, non-overlapping fields by ImageJ (US National Institutes of Health, Bethesda, MD, USA) software at a ×200 magnification in a blind manner by two independent pathologists.

### 4.6. ELISA for Inflammatory Cytokines in the Intestinal Mucosa

Intestinal segments were collected and centrifuged at 14,000 rpm for 60 s at 4 °C. The supernatants were collected and stored at −80 °C until further use. A multiplex protein assay kit (Bio-Rad, Hercules, CA, USA) was used to measure the mucosal levels of Th1 (IFN-γ, IL-1β and TNF-α), Th2 (IL-4, IL-5 and IL-10), and proinflammatory cytokines (IL-6). The results are expressed as pg/mL total protein.

### 4.7. Evaluation of MC Count

MC counts were evaluated by toluidine blue staining. All sections were stained with toluidine blue as previously described [31]. Mast cells were counted using an Olympus (Tokyo, Japan) BX43 microscope equipped with a 40× lens and 22 mm diameter oculars with a resulting high-power field (HPF) area measuring 0.237 mm^2^ (i.e., multiply cells/HPF by 4.22 for cells/mm^2^) by two independent pathologists. The toluidine blue-positive cells were counted in four contiguous, non-overlapping fields and expressed as MC/HPF. Another pathologist tested the interobserver reproducibility of MCs by comparing the counts. It was also checked whether the area of the sections examined by the pathologists were comparable.

### 4.8. Western Blot Analysis of Tryptase

MC-derived tryptase in mouse colonic tissues was detected by Western blotting. Protein extracts were separated by 10% sodium dodecyl sulfate-polyacrylamide gel electrophoresis and transferred to nitrocellulose membranes. The membranes were incubated with an anti-MC-derived tryptase antibody (Santa Cruz Biotechnology, Dallas, TX, USA) for the target molecule or anti-β-actin antibody (Santa Cruz Biotechnology, Dallas, TX, USA) for reference. After development, proteins were visualized with a ChemiDoc imaging system (Bio-Rad), and the protein bands were analyzed using ImageJ (US National Institutes of Health, Bethesda, MD, USA).

### 4.9. Statistical Analysis

Data were analyzed using GraphPad Prism software (San Diego, CA, USA). The AAD group was initially compared with the control group and then with the salbutamol group. All values are expressed as the mean ± standard error of the mean. Differences were considered statistically significant at *p* < 0.05. 

## 5. Conclusions

In conclusion, our study demonstrated that AAD induction elicited an inflammatory response in the intestine of mice, similar to that observed in IBS. To the best of our knowledge, no previous study has demonstrated an intestinal immune response during allergic reactions in the airways. This study suggests an association between IBS and AAD. Our study also indicates that in patients with IBS with such allergic components, treating AAD may improve the inflammatory response in the intestines. 

## Figures and Tables

**Table 1:** Colonic changes, including the measured colon length, colon weight, stool score, were analyzed as described in Materials and Methods. Data are presented as a mean ± standard deviation (n = 10 per group, * *p* < 0.05 vs control, ANOVA).

Parameter	Control	AAD Group	Salbutamol Group
Colon length, cm	11.50 ± 0.27	9.171 ± 0.22 *	11.40 ± 0.29
Colon weight, g	0.491 ± 0.014	0.498 ± 0.015	0.479 ± 0.018
Stool score	0.2 ± 0.133	1.3 ± 0.26 *	0.5 ± 0.167

## Data Availability

The datasets in this study are available from the corresponding author on reasonable request.

## Supplementary Materials

The following are available online at https://www.mdpi.com/article/10.3390/ijms23010181/s1.
