# Supplementary material for: Colonic Mucosal Immune Activation in Mice with Ovalbumin-Induced Allergic Airway Disease: Association between Allergic Airway Disease and Irritable Bowel Syndrome
Source: Int J Mol Sci. 2021 Dec 24;23(1):181. doi: 10.3390/ijms23010181 (PMC8745416; doi:10.3390/ijms23010181)
Supplement: Supplementary file 1 [file ijms-23-00181-s001.zip › ijms-1483837-supplementary.pdf]

Supplementary information

Title: Colonic Mucosal Immune Activation in Ovalbumin-Induced Allergic Airway Disease Mice: Association Between Allergic Airway Disease and Irritable Bowel Syndrome

Supplementary Table S1.

Primer and Probe Sequences for Murine Chemokines and Other Immune-Related Factors

Sequence (5′–3′)		
**IL-1β**	FW	CCTTCCAGGATGACATGA
	RV	TGAGTCACAGAGGATGGGCTC
**IFN-γ**	FW	TCAAGTGGCATAGATGTGGAAGAA
	RV	TGGCTCTGCAGGATTTTCATG
**IL-4**	FW	ACAGGAGAAGGGACGCCAT
	RV	GAAGCCCTACAGACGAGCTCA
**IL-5**	FW	AGCACAGTGGTGAAAGAGACCTT
	RV	TCCAATGCATAGCTGGTGATTT
**IL-6**	FW	GAGGATACCACTCCCAACAGACC
	RV	AAGTGCATCATCGTTGTTCATACA
**IL-10**	FW	GGTTGCCAAGCCTTATCGGA
	RV	ACCTGCTCCACTGCCTTGCT
**GAPDH**	FW	CATCCACTGGTGCTGCCAAGGCTGT
	RV	ACAACCTGGTCCTCAGTGTAGCCCA
